# The therapeutic effects of curcumin on polycystic ovary syndrome by upregulating PPAR-γ expression and reducing oxidative stress in a rat model

**DOI:** 10.3389/fendo.2024.1494852

**Published:** 2024-11-20

**Authors:** Wei Zhang, Cong Peng, Lei Xu, Yutai Zhao, Chaolin Huang, Ling Lu

**Affiliations:** Department of Gynecology and Obstetrics, Clinical Medical College and The First Affiliated Hospital of Chengdu Medical College, Chengdu, China

**Keywords:** curcumin, polycystic ovary syndrome, PPAR-γ, oxidative stress, reactive oxygen species

## Abstract

**Objective:**

Polycystic ovary syndrome (PCOS) is a prevalent endocrine and metabolic disorder that impacts 8-13% of women in their reproductive years. However, the drugs commonly used to treat PCOS are often prescribed off-label and may carry potential side effects. This study aimed to investigate the therapeutic effects of curcumin in a PCOS rat model.

**Materials and methods:**

A PCOS rat model was established through daily subcutaneous injection of 60 mg/kg body weight of dehydroepiandrosterone (DHEA) for 21 days. The PCOS rats received a daily intragastric dose of 50 mg/kg body weight of curcumin for another 21 days. Ovarian morphological changes, estrous cycle changes, and hormone level changes were measured to evaluate the therapeutic effectiveness of curcumin in PCOS rats. Oxidative stress markers in the ovaries were analyzed to explore the mechanisms of curcumin in PCOS rats.

**Results:**

This study demonstrated that curcumin alleviated insulin resistance and significantly reduced serum levels of estradiol (*p* = 0.02), luteinizing hormone (*p* = 0.009), testosterone (*p* = 0.003), and the LH/FSH ratio (*p* = 0.008) in PCOS rats. Curcumin also restored normal ovarian morphology and the estrous cycle in these rats. Furthermore, curcumin treatment significantly decreased levels of oxidative stress markers, including malondialdehyde (*p* = 0.004) and reactive oxygen species (*p* = 0.005), while increasing antioxidant levels such as superoxide dismutase (*p* = 0.04), glutathione peroxidase (*p* = 0.002), and glutathione (*p* = 0.02) in ovarian tissues. Additionally, curcumin significantly upregulated PPAR-γ in the ovarian tissues of PCOS rats.

**Conclusion:**

This study demonstrates that curcumin effectively restores ovarian morphology, hormone levels, and estrous cycles in PCOS rats. These effects may be linked to its ability to reduce oxidative stress in ovaries via the upregulation of PPAR-γ. Curcumin shows promise as a potential drug for the treatment of PCOS.

## 1 Introduction

Polycystic ovary syndrome (PCOS) is a prevalent endocrine and metabolic disorder that impacts 8-13% of women in their reproductive years (1, 2). It is characterized by various clinical manifestations, including hyperandrogenism, irregular menstruation, anovulation, infertility, and polycystic ovaries (3, 4). PCOS patients face an elevated risk of developing various diseases (5–7). Although the exact cause of PCOS is not fully understood, it is believed to result from a complex interplay of genetic, lifestyle, and environmental factors. Research suggests that hyperandrogenemia, inflammation, and oxidative stress are key contributors to the pathophysiology of PCOS (8, 9).

Oxidative stress plays an essential role in the development and progression of PCOS and its associated complications (10, 11). Oxidative stress arises when the generation of reactive oxygen species (ROS) surpasses the body’s ability to defend itself with antioxidants (12, 13). Key components involved in defending against oxidative stress include the antioxidant molecule glutathione and the enzymes superoxide dismutase and glutathione peroxidase (14, 15). Peroxisome proliferator-activated receptor gamma (PPAR-γ) is a nuclear receptor involved in various biological processes, including mitochondrial oxidative reactions, fatty acid oxidation, and glucose metabolism (16, 17). Research indicates that PPAR-γ enhances the expression of superoxide dismutase and glutathione peroxidase, thereby strengthening antioxidant defenses (18, 19). Additionally, PPAR-γ has been shown to reduce insulin resistance in PCOS (20, 21). These findings indicate that it could be a promising therapeutic target for treating PCOS.

Curcumin, a yellow polyphenol derived from the rhizomes of turmeric, a tropical plant native to Southeast Asia, is known for its potent anti-inflammatory and antioxidant properties (22). The antioxidant capacity of curcumin is underscored by its role in upregulating the expression of enzymes such as superoxide dismutase and glutathione peroxidase (22, 23). Although curcumin’s anti-inflammatory and antioxidant effects are well-documented, it remains unclear whether these mechanisms provide therapeutic benefits for PCOS, a condition characterized by chronic inflammation and oxidative stress. The drugs commonly used to treat PCOS are often prescribed off-label and may carry potential side effects. This study aims to assess the therapeutic potential of curcumin in managing PCOS and to explore the molecular mechanisms underlying its effects. The findings of this study may contribute to the development of new strategies for the treatment of PCOS.

## 2 Materials and methods

### 2.1 Experimental animals and treatments

The experimental protocols were approved by the Ethics Committee of The First Affiliated Hospital of Chengdu Medical College (Approval No. 2124131621). Eighteen female Sprague-Dawley (SD) rats, aged 6 weeks and weighing 174 ± 8 g, were kept at our laboratory animal center at a temperature of 22 ± 2°C with a 12-hour light/dark cycle. The rats were free to get food and water. Following a one-week acclimatization period, the rats were randomly divided into a control group (n = 6) and an experimental group (n = 12).

The experimental group was administered a daily subcutaneous injection of 60 mg/kg body weight of dehydroepiandrosterone (DHEA; Sigma-Aldrich, Shanghai, China) dissolved in 100 μl sesame oil for 21 days to induce a PCOS model according to the manufacturer’s instructions and previously published studies (24, 25). The control group was subcutaneously injected with the same amount of vehicle sesame oil. The successful establishment of the PCOS model was confirmed based on established criteria (26). Subsequently, the experimental group was further divided into two subgroups: the PCOS group (n = 6) and the PCOS-curcumin group (n = 6). The PCOS-curcumin group received a daily intragastric dose of 50 mg/kg body weight of curcumin powder (Product No. PHR2209, Sigma-Aldrich, Shanghai, China) suspended in 200μl purified water lasted for 21 days. The control PCOS rat group received 200μl purified water by gavage. The experimental group was administered DHEA continuously for another 21 days while treated with curcumin.

### 2.2 Estrous cycle determination

Vaginal smears were collected daily at 09:00 AM, air-dried on glass slides, and stained with 0.1% methylene blue (Sigma, Chengdu, China). The slides were evaluated under a microscope (BX41, Olympus), and images were captured. The phases of the estrous cycle were identified using vaginal cytology: a predominance of nucleated epithelial cells indicated the proestrus stage, the presence of mostly cornified squamous epithelial cells signified the estrus stage, a mix of cornified squamous epithelial cells and leukocytes characterized the metestrus stage, and a predominance of leukocytes marked the diestrus stage (26).

### 2.3 Morphological evaluation of ovaries

The mice were euthanized and the ovaries were carefully dissected, fixed in 4% paraformaldehyde at 4°C overnight, and then embedded in paraffin blocks. The paraffin blocks were cut into 4 μm thick serial sections and stained with hematoxylin and eosin (H&E) according to standard histological examination protocols. The morphology of ovaries and the number of follicular cysts were evaluated under a microscope.

### 2.4 Serum analysis

To avoid the impact of different estrous cycle stages on the levels of serum hormones, blood samples were collected at diestrus from the caudal veins of rats and centrifuged at 2000 g for 10 minutes to separate the serum. The serum levels of luteinizing hormone (LH), follicle-stimulating hormone (FSH), estradiol (E2), and testosterone (T) were measured using commercial ELISA kits purchased from Cusabio Technology LLC, the product numbers were CSB-E12654r, CSB-E06869r, CSB-E05110r, and CSB-E05100r, respectively. Each serum sample was tested in triplicates. Insulin resistance index (HOMA-IR) was calculated by the formula of fasting insulin (FIns, μU/ml) × fasting blood glucose (FBG, mmol/L)/22.5 as described in the previously published study (27).

### 2.5 Western blot analysis

Proteins were extracted from ovarian tissues, and their concentrations were determined using a bicinchoninic acid (BCA) protein quantification kit (Beyotime Biotechnology Inc., Shanghai, China). Each well was loaded with 15 μg of protein, which was then separated on a 10% sodium dodecyl sulfate-polyacrylamide electrophoresis (SDS-PAGE) gel and transferred onto polyvinylidene fluoride (PVDF) membranes. The membranes were blocked with 1.5% skimmed milk for one hour, followed by incubation with rabbit recombinant primary antibodies of anti-PPAR-γ (1:1000 dilution, ab316981, Abcam, Shanghai, China) and anti-GAPDH (1:1000 dilution, ab181602, Abcam, Shanghai, China) overnight at 4 °C. After three 15-minute washes with phosphate-buffered saline with tween-20 (PBST), the membranes were incubated with horseradish peroxidase-conjugated goat anti-rabbit IgG secondary antibody (1:2000 dilution, ab205718, Abcam, Shanghai, China) for 2 hours at room temperature. The protein bands on the PVDF membranes were then visualized using an enhanced chemiluminescence (ECL) kit (Thermo Fisher Scientific Inc., Shanghai, China). The quantitation of the proteins was determined by Image Lab 6.0 software.

### 2.6 Oxidative stress index analyses

The mice were euthanized, and ovarian tissues were harvested for oxidative stress index analyses. Oxidative stress markers, including reactive oxygen species (ROS) and malondialdehyde (MDA), were measured using the Reactive Oxygen Species Detection Assay Kit (ab186027, Abcam, Shanghai, China) and Lipid Peroxidation (MDA) Assay Kit (ab233471, Abcam, Shanghai, China), respectively. Antioxidant markers, including superoxide dismutase (SOD), glutathione peroxidase (GPx), and Glutathione (GSH), were assessed using the Superoxide Dismutase Activity Assay Kit (ab65354, Abcam, Shanghai, China), Glutathione Peroxidase Assay Kit (ab102530, Abcam, Shanghai, China), and Glutathione Assay Kit (ab65322, Abcam, Shanghai, China), respectively. All assays were performed according to the manufacturer’s instructions.

### 2.7 Statistical analysis

Statistical analyses were performed using SPSS 33.0 software. An unpaired Student’s t-test was used to compare differences between the two groups. A P-value of less than 0.05 was considered statistically significant.

## 3 Results

### 3.1 DHEA induced the PCOS model in rats

**Figure 1:**
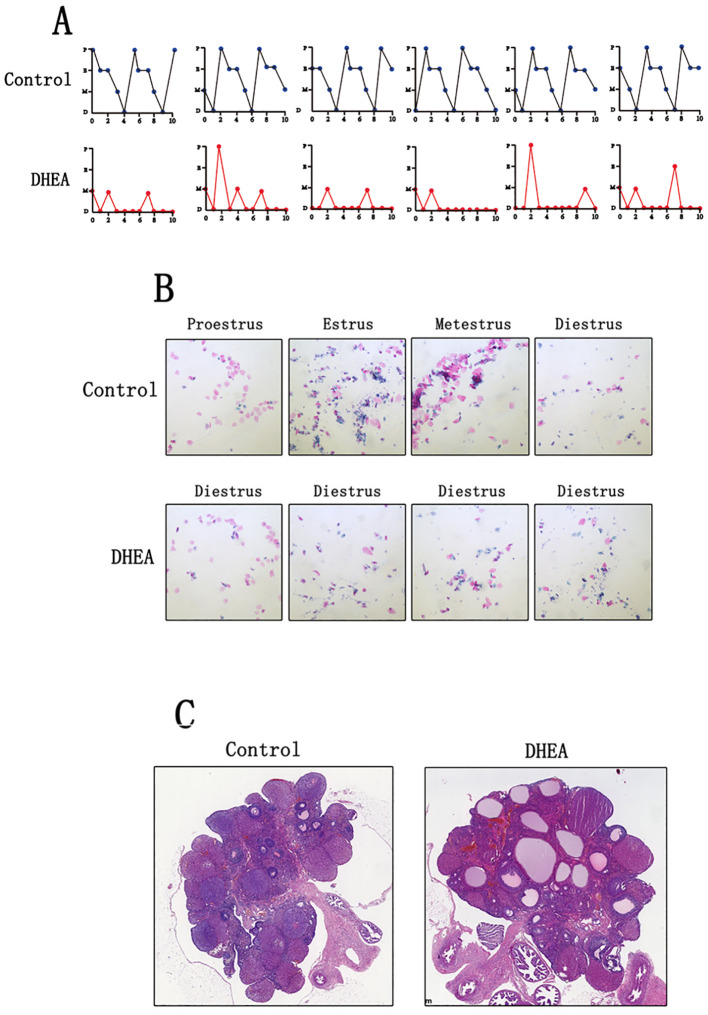
DHEA induced the PCOS model in rats (Control group: n=6, DHEA group: n=12). **(A)** the estrous cycle of normal rats and PCOS model rats (P: proestrus stage; E: estrus stage; M: metestrus stage; D: diestrus stage). **(B)** The vaginal smears of normal rats and PCOS model rats. **(C)** the ovarian morphology of normal rats and PCOS model rats.

Normal control rats exhibit regular sequential estrous cycles that include the proestrus stage, estrus stage, metestrus stage, and diestrus stage. However, DHEA-treated rats didn’t exhibit regular sequential estrous cycles, their estrous cycles were disrupted and stayed in the diestrus phase most of the time (**Figure 1A**). Vaginal smears revealed a predominance of leukocytes, with few keratinized epithelial cells observed, indicating anovulation in DHEA-treated rats (**Figure 1B**). Pathological examination showed an increased number of follicles with cystic dilation in the DHEA-treated rats, whereas the control group displayed a normal ovarian morphology (**Figure 1C**).

**Figure 2:**
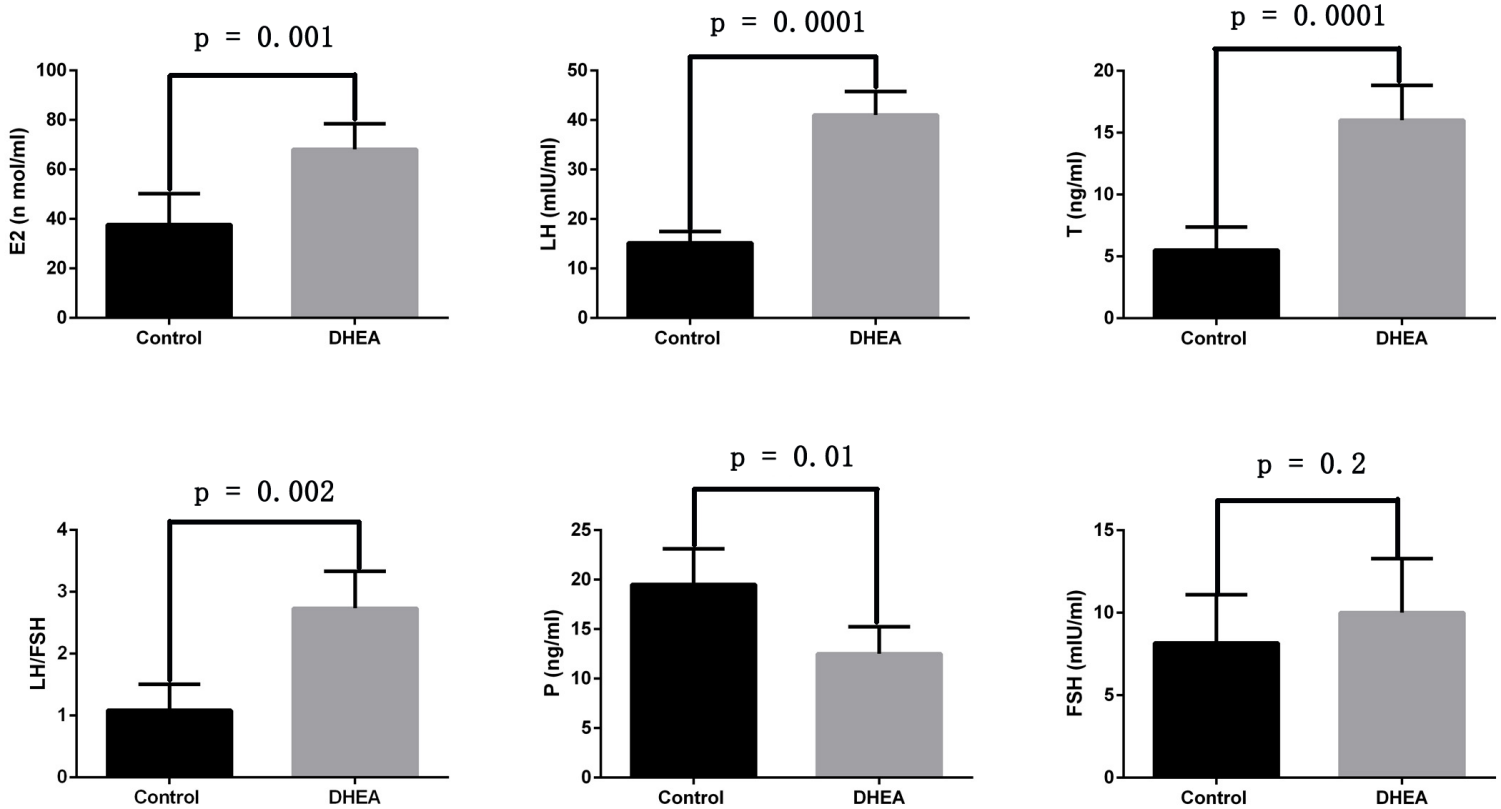
The sex hormone changes of normal rats and PCOS model rats (Control group: n=6, DHEA group: n=12). E2: estradiol; LH: luteinizing hormone; T: testosterone; P: progesterone; FSH: follicle-stimulating hormone.

Additionally, we assessed the serum levels of sex hormones. Compared to the control group, the DHEA-treated rats showed significantly elevated levels of estradiol (*p* = 0.001), luteinizing hormone (*p* = 0.0001), testosterone (*p* = 0.0001), and an increased LH/FSH ratio (*p* = 0.002). In contrast, progesterone levels (*p* = 0.01) were notably decreased. However, the two groups have no statistical difference in follicle-stimulating hormone levels (*p* = 0.2) (**Figure 2**). These findings suggested that we successfully established a PCOS rat model.

### 3.2 Curcumin alleviated sex hormone disorders and insulin resistance in PCOS rats

**Figure 3:**
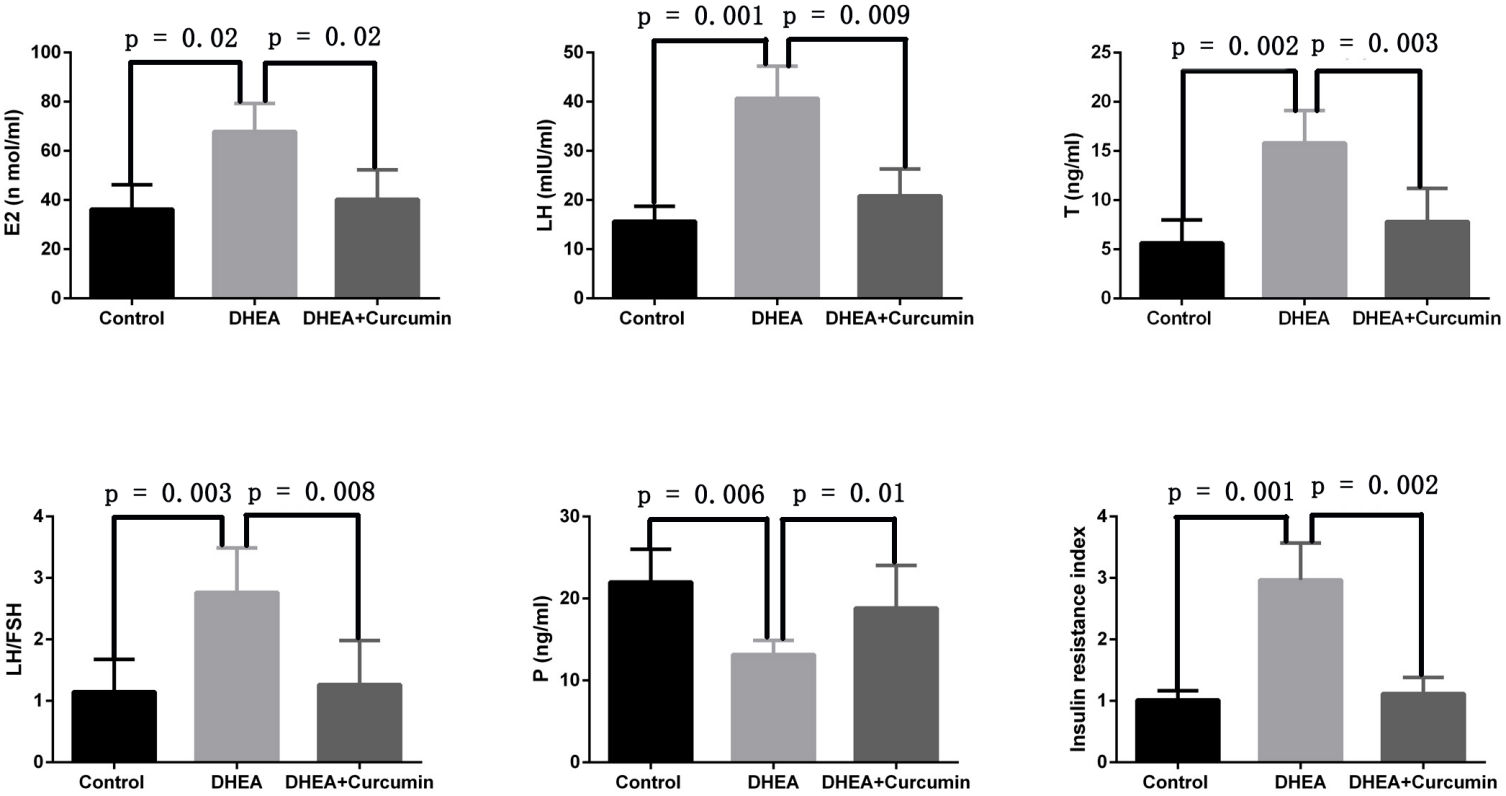
Curcumin alleviated sex hormone disorders and insulin resistance in PCOS rats (Control group: n=6, DHEA group: n=6, DHEA + Curcumin group: n=6). E2: estradiol; LH: luteinizing hormone; T: testosterone; P: progesterone.

We evaluated curcumin’s effect on the sex hormone imbalances associated with PCOS. The findings indicated that curcumin significantly reduced serum levels of estradiol (*p* = 0.02), luteinizing hormone (*p* = 0.009), testosterone (*p* = 0.003), and the LH/FSH ratio (*p* = 0.008) in PCOS rats. However, curcumin significantly increased serum progesterone levels (*p* = 0.01) in these rats. Additionally, curcumin alleviated insulin resistance in PCOS rats (**Figure 3**).

### 3.3 Curcumin restored estrous cycle and ovarian morphology in PCOS rats

**Figure 4:**
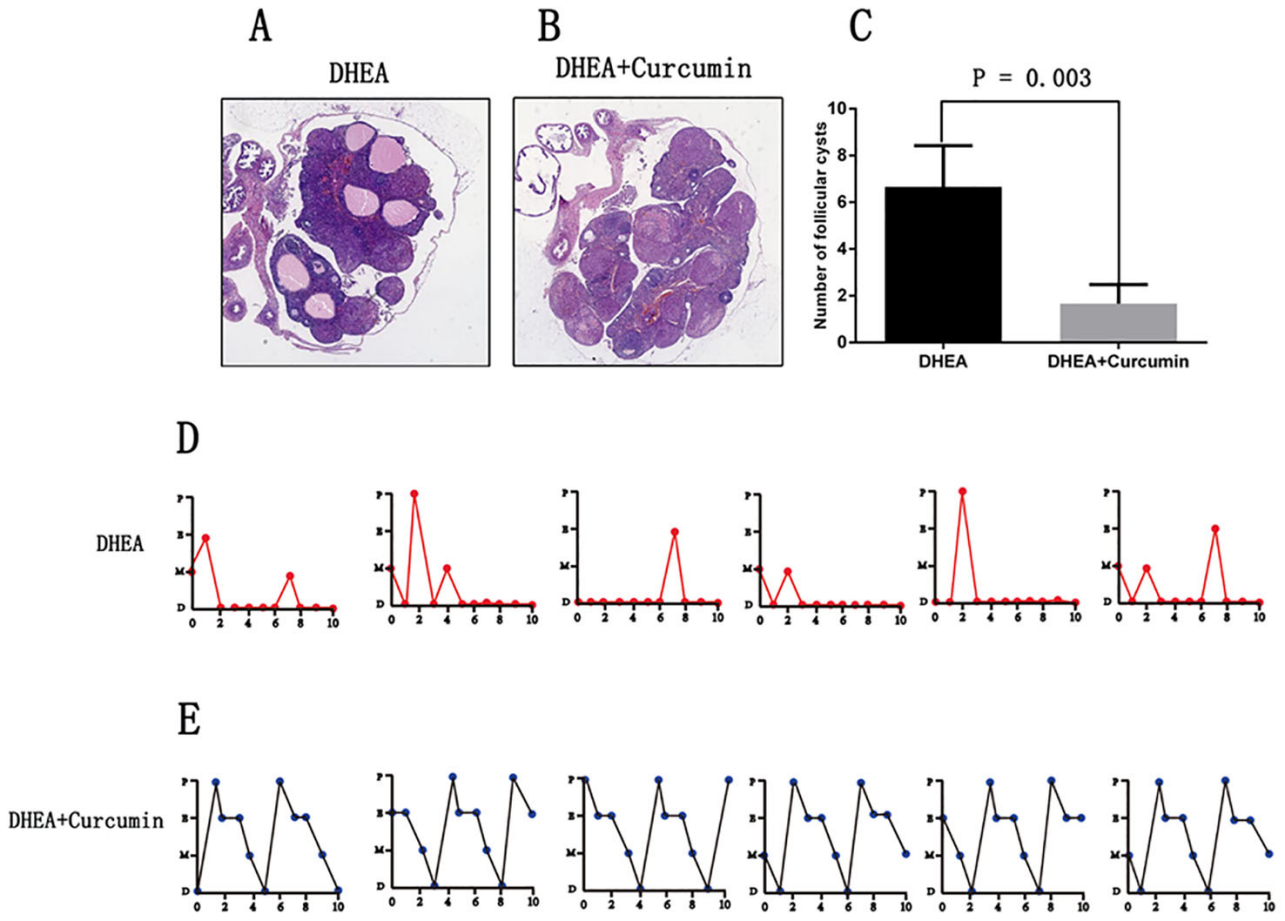
Curcumin restored estrous cycle and ovarian morphology in PCOS rats (DHEA group: n=6, DHEA + Curcumin group: n=6). **(A)** The ovarian morphology of PCOS model rats. **(B)** The ovarian morphology of curcumin-treated PCOS model rats. **(C)** The number of follicular cysts in each ovary. **(D)** The estrous cycle of PCOS model rats. **(E)** the estrous cycle of curcumin-treated PCOS model rats. P: proestrus stage; E: estrus stage; M: metestrus stage; D: diestrus stage.

The ovarian morphology of PCOS rats showed polycystic changes (**Figure 4A**). After the curcumin treatment, the PCOS rats showed multiple corpus luteum and no polycystic changes (**Figure 4B**). After the curcumin treatment, the number of follicular cysts decreased significantly in the PCOS rat model (**Figure 4C**). The estrous cycles of PCOS rats were disrupted and stayed in the diestrus stage most of the time (**Figure 4D**). However, curcumin treatment restored the normal estrus cycles (**Figure 4E**).

### 3.4 Curcumin suppressed oxidative stress and upregulated PPAR-γ in PCOS rats

**Figure 5:**
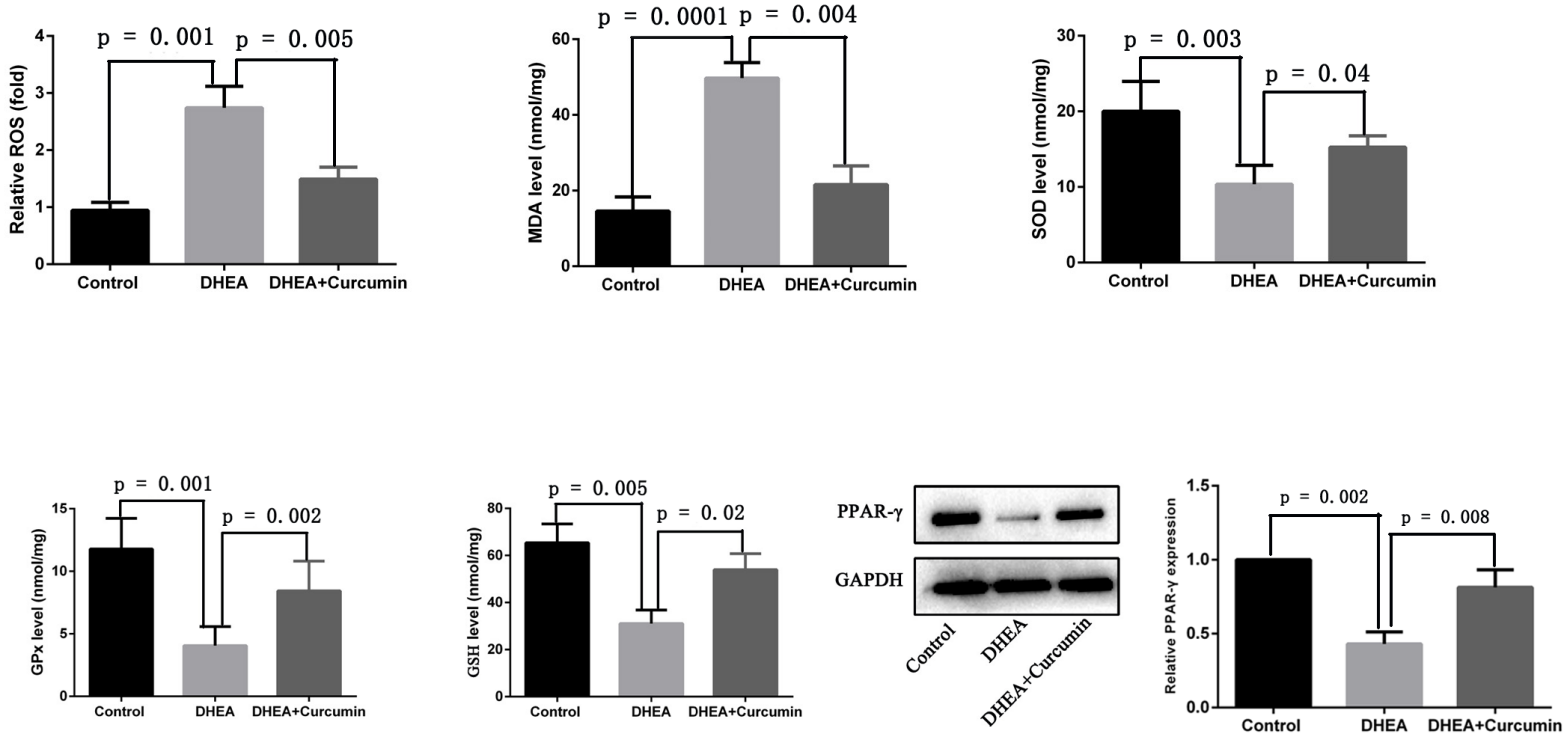
Curcumin reduced oxidative stress and upregulated PPAR-γ in PCOS rats (Control group: n=6, DHEA group: n=6, DHEA + Curcumin group: n=6). ROS: oxygen species; MDA: malondialdehyde; SOD: superoxide dismutase; GPx: glutathione peroxidase; GSH: glutathione.

To assess the effectiveness of curcumin on oxidative stress in PCOS rats, we measured reactive oxygen species (ROS) levels and the levels of enzymes critical for maintaining redox balance. The results showed that PCOS rats exhibited significantly increased levels of oxidative indicators, including ROS and malondialdehyde, along with decreased levels of antioxidants such as superoxide dismutase, glutathione peroxidase, and glutathione in ovarian tissues. However, treatment with curcumin in PCOS rats significantly reduced ROS (*p* = 0.005) and malondialdehyde (*p* = 0.004) levels while increasing superoxide dismutase (*p* = 0.04), glutathione peroxidase (*p* = 0.002), and glutathione (*p* = 0.02) levels in ovarian tissues (**Figure 5**). Additionally, we observed that the expression of PPAR-γ was significantly decreased in the ovarian tissues of PCOS rats, but curcumin treatment significantly upregulated PPAR-γ expression (**Figure 5**).

## 4 Discussion

PCOS is a prevalent endocrine and metabolic disorder that impacts 8-13% of women in their reproductive years (1, 2). While medications such as letrozole, clomiphene, and metformin are commonly used to manage PCOS symptoms, it is important to note that these drugs are often prescribed off-label and may carry potential side effects (28). As a result, there has been significant interest in developing new and more effective treatments for PCOS. Recently, studies have reported that traditional Chinese medicine and food-derived ingredients, such as allium extracts, soy isoflavones, and crocetin, have emerged as promising effectiveness for PCOS management (29, 30). These findings suggested that botanical ingredients have the potential for further advancements in PCOS treatment options.

Curcumin has been extensively studied for its diverse biological activities, including potent anti-inflammatory, antioxidant, and anticancer properties (22, 31). This study demonstrated that curcumin alleviated insulin resistance and significantly reduced serum levels of luteinizing hormone (LH), testosterone (T), and the LH/FSH ratio in PCOS rats. Additionally, curcumin restored normal ovarian morphologies and estrous cycles in these PCOS rats. Previous studies suggested that oxidative stress is a key contributor to the development and progression of PCOS and its associated complications. The therapeutic effectiveness of curcumin in PCOS rats may be due to its antioxidant ability to reduce oxidative stress.

Previous research has identified curcumin as a potent antioxidant, with its antioxidant properties attributed to the hydroxyl group and methylene group within its β-diketone structure (22). Under normal physiological conditions, various antioxidants protect the body from the damaging effects of oxidative stress. Known antioxidants include glutathione, vitamin E, catalase, vitamin C, and glutathione peroxidase, superoxide dismutase (32). However, during oxidative stress, the levels of these antioxidants are reduced. In our study, PCOS rats exhibited significantly increased levels of oxidative indicators, including ROS and malondialdehyde, along with decreased levels of antioxidants such as glutathione, superoxide dismutase, and glutathione peroxidase in ovarian tissues. These findings suggest that the PCOS rats were in a state of oxidative stress. Treatment with curcumin in PCOS rats significantly reduced the levels of ROS and malondialdehyde while increasing the levels of glutathione, glutathione peroxidase, and superoxide dismutase in ovarian tissues. These findings demonstrate that curcumin has decreased oxidative stress in PCOS rats.

Curcumin mitigates oxidative stress by upregulating PPAR-γ expression, a nuclear receptor integral to the regulation of inflammation, glucose metabolism, and lipid balance (16, 17, 33). Our research demonstrated that PPAR-γ expression was significantly reduced in the ovarian tissues of PCOS rats, but treatment with curcumin significantly upregulated PPAR-γ expression. The precise mechanisms by which curcumin upregulates PPAR-γ expression are still unclear. Two possible scenarios exist: curcumin may bind to a specific receptor, forming a complex that promotes PPAR-γ upregulation, or curcumin might act directly on transcription factors to promote gene expression of PPAR-γ. Despite this uncertainty, many studies have linked curcumin’s ability to reduce oxidative stress to PPAR-γ upregulation (34, 35). Furthermore, recent research has shown that curcumin exerts antitumor effects in pancreatic cancer by inhibiting cell proliferation, angiogenesis, and the NF-κB signaling pathway (36). Therefore, it is plausible to propose that curcumin reduces oxidative stress in PCOS rats by upregulating PPAR-γ expression and subsequently inhibiting the NF-κB signaling pathway.

Previous studies have shown that curcumin can reduce serum testosterone and luteinizing hormone levels while alleviating insulin resistance in PCOS rat models (37). Our research builds on these findings by demonstrating that curcumin also normalizes the estrous cycles and restores ovarian morphologies in PCOS rats. Additionally, our study provides novel insights by highlighting the crucial role of PPAR-γ in mediating curcumin’s therapeutic effects on PCOS.

The study has several limitations that should be acknowledged. First, the therapeutic effects of curcumin on PCOS rats were investigated only *in vivo*, so further *in vitro* studies are needed to understand the underlying mechanisms. Second, the research was conducted using 6-week-old rats, which do not represent the entire reproductive age population; further studies involving rats of varying ages are needed to confirm these findings. Finally, since this research was conducted using a PCOS rat model, cautions are needed before generalizing the findings to humans. Further randomized clinical trials are required to validate these findings in human populations.

## 5 Conclusions

In summary, this study demonstrates that curcumin effectively restores ovarian morphologies, hormone levels, and estrous cycles in PCOS rats. The findings suggest that curcumin’s therapeutic effects in PCOS may be linked to its ability to reduce oxidative stress in ovaries through the upregulation of PPAR-γ. Curcumin shows promise as a potential drug for the treatment of PCOS. However, further studies are needed to confirm these findings and to understand their underlying mechanisms.

## Data Availability

The original contributions presented in the study are included in the article/supplementary material. Further inquiries can be directed to the corresponding authors.
